# Mobile-phone and handheld microscopy for neglected tropical diseases

**DOI:** 10.1371/journal.pntd.0005550

**Published:** 2017-07-06

**Authors:** Jason Rajchgot, Jean T. Coulibaly, Jennifer Keiser, Jürg Utzinger, Nathan C. Lo, Michael K. Mondry, Jason R. Andrews, Isaac I. Bogoch

**Affiliations:** 1 Department of Medicine, University of Toronto, Toronto, Canada; 2 Unité de Formation et de Recherche Biosciences, Université Félix Houphouët-Boigny, Abidjan, Côte d’Ivoire; 3 Centre Suisse de Recherches Scientifiques en Côte d’Ivoire, Abidjan, Côte d’Ivoire; 4 Swiss Tropical and Public Health Institute, Basel, Switzerland; 5 University of Basel, Basel, Switzerland; 6 Division of Infectious Diseases and Geographic Medicine, Stanford University School of Medicine, Stanford, California, United States of America; 7 Division of Epidemiology, Stanford University School of Medicine, Stanford, California, United States of America; 8 Department of Mechanical Engineering, Stanford University, Stanford, California, United States of America; 9 Divisions of General Internal Medicine and Infectious Diseases, Toronto General Hospital, Toronto, Canada; Task Force for Child Survival and Developmentorce for Global Health, UNITED STATES

Diagnostic laboratory infrastructure in low- and middle-income countries (LMICs) is notoriously scarce, with limited resources typically clustered in urban settings where they are inaccessible to much of the population [1]. One attempt to mitigate these issues is the development and implementation of handheld and mobile-phone microscope technologies. These approaches aim to deliver high-quality laboratory diagnostic capability to resource-constrained settings by bringing the diagnostics to the people rather than transferring people or clinical specimens to distant laboratories. The goal is to provide robust, inexpensive, and accurate devices that can be utilized at the point of care in the most austere of settings. Despite a wealth of technological innovation in this field meeting many of these criteria, there remain key challenges in implementing mobile-microscopy programs in resource-constrained environments [2]. Here, we review some of the recent approaches, discuss their strengths and limitations, and offer considerations for a way forward in bringing mobile microscopy to communities in need.

While there have been many publications over the past decade reporting on portable or mobile-phone microscopes, comparably few studies have been performed in which real clinical specimens were tested under field conditions. Table 1 summarizes recent field studies in which the accuracy of mobile microscopes for detection of neglected tropical diseases (NTDs) in clinical specimens was evaluated. One early, very low-cost device utilized a glass ball lens mounted to the camera lens of a mobile phone for magnification. This approach was rather cumbersome to use, given the small field of view and movement of the ball lens, which resulted in poor image quality and insufficient diagnostic sensitivity and specificity for soil-transmitted helminth infections [3]. The paper-based Foldscope is an inexpensive and lightweight device, also utilizing a ball lens for magnification, as well as a battery-powered LED for illumination [4]. When mounted to a mobile phone, the Foldscope demonstrated low sensitivity (although it had high specificity) for the diagnosis of *Schistosoma haematobium* infection, possibly due to the small field of view and challenges with slide navigation under the lens [5]. The reversed-lens CellScope is a lightweight plastic attachment with an embedded lens that harnesses the light source from a mobile phone. This device is manually maneuvered over a sample and when tested on *S*. *haematobium* samples, it demonstrated modest sensitivity with excellent specificity [5].

**Table 1:** Recent field validation studies involving mobile and handheld light microscopy for neglected tropical infections in clinical specimens in endemic settings.

Device	Organism detected	Sample size	Sensitivity/specificity* (%)	PPV/NPV (%)	Reference
**Ball lens mounted to mobile-phone camera**	*Ascaris lumbricoides*, *Trichuris trichiura*, and hookworm	199	69.4/61.5	92.3/23.2	Bogoch et al. 2013 [3]
**Mobile phone–mounted Foldscope**	*Schistosoma haematobium*	49	55.9/93.3	95.0/48.3	Ephraim et al. 2015 [5]
**Reversed-lens CellScope**	*S*. *haematobium*	49	67.6/100.0	100.0/57.7	Ephraim et al. 2015 [5]
**Newton Nm1 with mobile phone attached**	*S*. *mansoni*	226	91.7/99.5	91.7/99.5	Coulibaly et al. 2016 [6]
**Newton Nm1 with mobile phone attached**	*S*. *haematobium*	226	81.1/97.1	94.8/88.6	Coulibaly et al. 2016 [6]
**CellScope Loa**	*Loa loa*	33	100/94		D’Ambrosio et al. 2015 [7]
**Newton Nm1 with mobile phone attached**	*Plasmodium falciparum*	223	80.2/100.0	100.0/65.6	Coulibaly et al. 2016 [8]

*Sensitivity and specificity of mobile-phone or handheld microscopes for the diagnosis of *S*. *mansoni*, *S*. *haematobium*, and soil-transmitted helminths was compared to conventional light microscopy.

**Abbreviations:** NPV, negative predictive value; PPV, positive predictive value

The results from the aforementioned technologies were obtained by expert microscopists under near-ideal conditions. However, a critical issue is understanding how well these devices will perform in routine clinical or public-health settings. Only a few mobile-phone microscopes have been evaluated in “real world” settings by individuals who would use these devices in their routine practice. Coulibaly and colleagues trained local laboratory technicians how to use a reversed-lens CellScope and the Newton Nm1 handheld microscope with a mobile-phone attachment as part of a community-based screening program for schistosomiasis in Côte d’Ivoire [6]. Local laboratory technicians successfully used these devices, and the Newton Nm1 microscope demonstrated diagnostic sensitivities for *S*. *mansoni* and *S*. *haematobium* eggs of 91.7% and 81.1%, respectively, and specificities of 99.5% and 97.1%, respectively, while the CellScope demonstrated sensitivities for *S*. *mansoni* and *S*. *haematobium* of 50.0% and 35.6%, respectively, and specificities of 99.5% and 100%, respectively [6]. A version of the CellScope is currently under revision to enable improved sensitivity, as microscopists had some challenges manually navigating the device around a slide.

Another device, the CellScope Loa, was tested to identify and quantify *Loa loa* microfilaremia in Cameroon by mobilizing a reversed-lens microscope attachment in combination with a custom application [7]. The device uses video microscopy and computer vision to identify motion of *L*. *loa* microfilaria and can process a blood film within 2 min. In an initial test of 33 patients, the device achieved a sensitivity of 100% and specificity of 94% when compared to conventional light microscopy [7]. It is currently being evaluated in a larger cohort. Lastly, Ivorian laboratory technicians used the Newton Nm1 handheld microscope with a mobile-phone attachment to detect *Plasmodium falciparum* in a community-based screening program in rural Côte d’Ivoire, with sensitivity and specificity of 80.2% and 100.0%, respectively, compared to “gold” standard microscopy [8]. These studies highlight that novel devices can be used in routine public health practice in resource-constrained settings and ongoing studies are currently building on this early success.

The ubiquity of mobile phones in LMICs and the growing quality of and access to mobile networks across the globe continue to make mobile-phone microscopy a compelling technology [2,9]. In addition, the impressive capabilities of mobile-phone microscopes continue to be demonstrated in laboratories with plans for validation and scale in real world settings. Recent developments include imaging of single DNA molecules [10] and computer-vision and machine-learning technology for the automated detection and quantification of pathogens [11]. The ability of these devices to connect with databases and global positioning system (GPS) devices provides compelling potential public health, clinical, and research applications (Table 2).

**Table 2:** Advantages and disadvantages of handheld and mobile-phone microscopy.

Advantages	Disadvantages
• Devices are portable and can be easily transported to rural and remote settings.	• Portable imaging technology continues to evolve and still requires design improvement to increase diagnostic sensitivity and user friendliness.
• Mobile phones are ubiquitous.	• Sample preparation is still required, as with conventional microscopy.
• Devices are battery powered and not affected by intermittent power outages.	• Diagnostic devices require further validation in clinical and public-health settings, and wider application across multiple pathogens will be necessary.
• Image-processing algorithms can enable automated diagnoses; global positioning systems and transmission features of phones can facilitate data sharing for better disease-burden estimates.	• Some devices remain an expensive option when compared to traditional light microscopy.

Microscopes for use in LMICs should be able to maintain appropriate sensitivity and specificity in rugged environments with variable sample quality. Current designs focus on usability by all members of the healthcare team. Ideally, microscopes would be designed with intuitive, foolproof operation in mind so that they could be used even by untrained individuals, much like the automatic external defibrillator. Similarly, sample preparation must be simplified such that diagnoses at the point of care are possible [12] and systems designed to ensure appropriate quality-control measures are adhered to. It is also important to note that initial manufacturing costs will likely be unimportant to the overall cost of microscope operation when factored over many years. Microscope development should focus on building robust, high-quality devices that can achieve high throughput. Consider this hypothetical scenario: a US$1 microscope can process 30 specimens per day, which equals 7,200 specimens over the course of a year, assuming a laboratory technician working 5 days a week for 48 weeks per year. Incorporating laboratory technician salary and associated costs (for example, US$4,000 per year) as well as the US$1 upfront device cost, the cost per specimen over a 10-year time span would be US$0.56. Using a similar framework, consider a microscope that initially costs US$500 but has higher throughput (e.g., larger field of view, easier slide manipulation, or image-detection software) and can process 40 specimens per day (96,000 samples over 10 years). In this same hypothetical 10-year time span with the annual laboratory technician salary of US$4,000, the cost per specimen would be only US$0.42, despite the higher up-front investment. It follows that, rather than focusing primarily on reducing instrument costs, products should be designed to ensure ease of use and rapid throughput of specimens. In addition, ultra-low-cost devices may have a significantly shorter life span and may not have the most reliable diagnostic operating characteristics compared to more expensive and more robust equipment.

One of the key barriers to implementation of mobile microscopy for NTDs is the need for slide preparation from blood, stool, urine, and tissue specimens. Many slide-preparation techniques require centrifugation or filtration as well as organism staining. There has been comparatively little work done on simple, low-cost, laboratory-free means for microscopy sample preparation, and yet, without solving this challenge, the potential of mobile microscopy for use in remote settings without laboratory infrastructure will not be realized.

An important consideration with any new technology is the potential barriers to implementation and scale. Currently, there is no set of standards that exists to ensure quality of these devices. These technologies will likely only last if they are properly maintained and functional, and healthcare practitioners will need to be trained to use a novel technology. Hence, there is a need for innovative manufacturing pathways and viable business models. Lastly, the devices must be shown to provide significant value to current models, such as ease of use, portability, and improving access to care.

Portable microscopes hold potential for expanding access to diagnosis for NTDs in resource-constrained settings. A number of innovative approaches have appeared in the literature, and early field testing has yielded promising results. Further work is needed to create sample-to-answer solutions that address current obstacles to sample preparation, object identification, and high-throughput use. Once these challenges are addressed, portable microscopy could yield timely information on the distribution and of disease in remote settings and transform the approach to treatment, control, surveillance, and elimination of NTDs at the point of care.
